# Prone positioning in severe ARDS requiring extracorporeal membrane oxygenation

**DOI:** 10.1186/s13054-020-03110-2

**Published:** 2020-07-08

**Authors:** Jonathan Rilinger, Viviane Zotzmann, Xavier Bemtgen, Carin Schumacher, Paul M. Biever, Daniel Duerschmied, Klaus Kaier, Peter Stachon, Constantin von zur Mühlen, Manfred Zehender, Christoph Bode, Dawid L. Staudacher, Tobias Wengenmayer

**Affiliations:** 1 Department of Medicine III (Interdisciplinary Medical Intensive Care), Medical Center, University of Freiburg, Faculty of Medicine, University of Freiburg, Freiburg, Germany; 2 Department of Cardiology and Angiology I, Heart Center Freiburg University, Faculty of Medicine, University of Freiburg, Hugstetterstr. 55, 79106 Freiburg, Germany; 3 Institute of Medical Biometry and Statistics, University Medical Center Freiburg, Faculty of Medicine, University of Freiburg, Freiburg, Germany

**Keywords:** ECMO, Extracorporeal membrane oxygenation, Prone positioning, Acute respiratory distress syndrome, Outcome

## Abstract

**Background:**

Prone positioning (PP) has shown to improve survival in patients with severe acute respiratory distress syndrome (ARDS). To this point, it is unclear if PP is also beneficial for ARDS patients treated with veno-venous extracorporeal membrane oxygenation (VV ECMO) support.

**Methods:**

We report retrospective data of a single-centre registry of patients with severe ARDS requiring VV ECMO support between October 2010 and May 2018. Patients were allocated to the PP group if PP was performed during VV ECMO treatment or the supine positioning group. VV ECMO weaning success and hospital survival were analysed before and after propensity score matching.

**Results:**

A total of 158 patients could be analysed, and 38 patients (24.1%) received PP. There were no significant differences in VV ECMO weaning rate (47.4% vs. 46.7%, *p* = 0.94) and hospital survival (36.8% vs. 36.7%, *p* = 0.98) between the prone and supine groups, respectively. The analysis of 38 propensity score matched pairs also showed no difference in hospital survival (36.8% vs. 36.8%, *p* = 1.0) or VV ECMO weaning rate (47.4% vs. 44.7%, *p* = 0.82). Hospital survival was superior in the subgroup of patients treated with early PP (cutoff < 17 h via Youden’s Index) as compared to late or no PP (81.8% vs. 33.3%, *p* = 0.02).

**Conclusion:**

In this propensity score matched cohort of severe ARDS patients requiring VV ECMO support, prone positioning at any time was not associated with improved weaning or survival. However, early initiation of prone positioning was linked to a significant reduction of hospital mortality.

## Background

In case of severe acute respiratory distress syndrome (ARDS), veno-venous extracorporeal membrane oxygenation (VV ECMO) support may be considered when lung-protective mechanical ventilation is not able to prevent hypoxia or hypercapnia [1–3]. Nevertheless, mortality of severe ARDS remains high—even with ECMO support. The EOLIA trial for instance showed a mortality rate of 35% in patients treated with ECMO compared to 46% in patients without ECMO support in very severe ARDS [2].

Moreover, several studies showed that prone positioning (PP) is able to improve survival in these critically ill patients [4, 5]. PP provides various positive effects on oxygenation and lung compliance [6, 7]. Furthermore, PP can reduce ventilator-induced lung injury [8] and is associated with less days on mechanical ventilation (MV) and shorter length of intensive care unit (ICU) stay [5].

Hence, PP might be beneficial for patients receiving ECMO support. It has been demonstrated that PP can be performed safely [9–13] during ECMO support and improves oxygenation and lung compliance [14]. So far, there is little evidence about the outcome of these patients. We performed a retrospective analysis of ARDS patients treated with PP during ECMO support at our centre.

## Methods

We report retrospective data of a single-centre registry of patients with severe ARDS treated with VV ECMO. All patients treated at the Interdisciplinary Medical Intensive Care Unit at the Medical Centre, University of Freiburg, Germany, between October 2010 and May 2018 were registered. Patient identity data derived from the registry were blinded, and the study plan was approved by the local ethics committee (EK-Freiburg 151/14).

### Study population

All patients suffered from severe ARDS. VV ECMO support was initiated in cases of severe hypoxic respiratory failure or CO_2_ retention despite of mechanical ventilation as suggested by the ELSO guidelines. Patients receiving PP during ECMO support were allocated to the prone group, whereas the remaining patients formed the supine group. PP before initiation of ECMO support did not influence the allocation of patients in one or the other group. Primary endpoints were successful ECMO weaning, and ICU and hospital survival. Successful ECMO weaning was defined as being free from ECMO and alive for at least 48 h after decannulation. Unsuccessful weaning was defined as the inability to explant the ECMO device because of persistent respiratory failure or death during ECMO support and the need for re-cannulation within 48 h. Moreover, ventilator settings of the first 10 days after ECMO initiation were analysed.

To compare the patients’ disease severity, the RESP [15], SOFA [16], and APACHE II scores [17] as well as the Horowitz index (PaO_2_/FiO_2_) were analysed.

### ECMO centre and ECMO management

Our institution features a 24/7 ECMO centre localised within a tertiary hospital with a 30-bed medical intensive care unit. Cannulations in our ECMO centre are performed by two experienced intensivists and a perfusionist in Seldinger’s technique without primary surgical cut down. All member of the ECMO team can be gathered within 30 min. Typical numbers for veno-arterial and veno-venous cannulations are 65 and 35 per year, respectively. There is a 24 h/7 days outreach team. For this research, only in-house cases were considered. As ECMO system, either SCPC (Sorin Centrifugal Pump Console, LivaNova, London, UK) or Cardiohelp (Maquet Getlinge Group, Rastatt, Germany) was used. Cannulation was predominantly performed with dual-lumen cannula (Avalon, Maquet, Rastatt, Germany). For patients without life-threatening bleeding, anticoagulation was provided by intravenous unfractionated heparin aiming at a partial thromboplastin time 1.5 times upper normal limit. The management of vasopressors and fluid therapy was driven by clinical judgement of the ECMO experienced intensivist in charge and has been reported earlier [18].

Treatment algorithms and standard operating procedures were subject to optimizations during the observational period, reflecting current state-of-the-art recommendations and scientific knowledge.

Controlled MV mode used at our institution mostly was biphasic positive airway pressure (BIPAP). In few patients, airway pressure release ventilation (APRV) was used, when considered beneficial. VV ECMO support was implemented in case of severe but potentially reversible respiratory failure, when lung-protective MV resulted in hypoxemia or hypercapnia. Lung-protective MV was defined as positive end expiratory pressure (PEEP) ≤ 15 cmH_2_O, plateau pressure ≤ 30 cmH_2_O, driving pressure ≤ 15 cmH_2_O, and FiO_2_ ≤ 50%. Cannulation was performed predominately jugulary using a dual-lumen cannula.

After initiation of the VV ECMO support, invasivity of MV was reduced and ECMO flow was adjusted aiming for a peripheral oxygen saturation of 85–90% and partial pressure arterial oxygen of approximately 50 mmHg, respectively. Typical ventilator settings were as follows: PEEP 15 cmH_2_O, plateau pressure 25 cmH_2_O, FiO_2_ 50%, and respiratory rate 10/min.

### Indications and performance of prone positioning during ECMO support

ARDS treatment was carried out according to the currently valid guidelines [19]. The decision on whether to perform PP in the individual case lays with the treating medical team’s judgement.

Prone positioning was done face down. Sedation for PP patients at our institution was titrated to preserve spontaneous breathing if possible. Neuromuscular blockade was not given on a routine basis for executing PP. However, in individual cases, especially in cases of strong respiratory drive and concerns about a self-inflicted lung injury [20], neuromuscular blocking agents were used.

### Statistical analysis

Summary results for categorical variables are presented as frequency and percentage. Results for numeric variables are presented as median with interquartile range (IQR). Fisher’s exact test and Pearson’s chi-squared test were used for analysing nominal variables. In dependence of normal distribution, Student’s *t* test or Mann-Whitney *U* test was performed for continuous variables.

Multivariate regression analysis was performed for univariate (dependent) predictors of hospital survival. Results are given as odds ratio [(OR), 95% confidence interval (CI)], and a *p* value of ≤ 0.05 was considered statistically significant. ROC analysis and Youden’s Index (Youden’s Index = Sensitivity + Specificity − 1) were used for reaching the optimal cutoff of survival-associated factors with highest discrimination of sensitivity and specificity.

Propensity score matching was performed using SPSS with a nearest neighbour matching algorithm using a calliper of 0.01. Matching was performed for age, sex, SOFA score, the duration of MV before ECMO, and performance of prior PP before ECMO. Cumulative incidences of 60-day mortality were calculated using competing risk regression (Fine and Gray method) with discharge alive considered a competing event [21]. Statistical calculations were performed using IBM SPSS statistics 25.0 (Armonk, NY: IBM Corp, 2017).

## Results

### Patients

A total of 158 patients with complete medical data could be analysed (age 54.5 (41.8–64.0) years, 67% male). The collective showed a relatively high rate of comorbidities, and this was especially true for immunosuppression (36%, Table 1).

**Table 1:** Baseline characteristics

	All (*n* = 158)	Prone (*n* = 38)	Supine (*n* = 120)	*p* value
**Demographics**				
Age (years)	54.5 (41.8–64.0)	51.5 (38.5–64.0)	55.5 (44.0–64.0)	0.549
Sex (male)	106 (67.1%)	28 (73.7%)	78 (65.0%)	0.321
BMI (kg/m^2^)	24.7 (22.9–28.8)	24.8 (24.0–28.7)	24.4 (22.9–28.9)	0.566
Underlying pulmonary disease	55 (34.8%)	12 (31.6%)	43 (35.8%)	0.631
COPD	11 (7.0%)	1 (2.6%)	10 (8.3%)	0.229
Asthma	10 (6.3%)	1 (2.6%)	9 (7.5%)	0.283
Lung fibrosis	18 (11.4%)	5 (13.2%)	13 (10.8%)	0.694
Cystic fibrosis	7 (4.4%)	2 (5.3%)	5 (4.2%)	0.775
LTOT	11 (7.0%)	1 (2.6%)	10 (8.3%)	0.229
Pulmonary hypertension	6 (3.8%)	0 (0.0%)	6 (5.0%)	0.160
Comorbidities				
Nicotine abuse	47 (29.7%)	9 (23.7%)	38 (31.7%)	0.348
Hypertension	49 (31.0%)	10 (26.3%)	39 (32.5%)	0.473
Diabetes mellitus	24 (15.2%)	6 (15.8%)	18 (15.0%)	0.906
CAD	20 (12.7%)	5 (13.2%)	15 (12.5%)	0.915
Chronic renal failure	12 (7.6%)	6 (15.8%)	6 (5.0%)	0.029
Chronic haemodialysis	2 (1.3%)	1 (2.6%)	1 (0.8%)	0.388
Liver cirrhosis/hepatitis	17 (10.8%)	3 (7.9%)	14 (11.7%)	0.513
Immunosuppression	57 (36.1%)	13 (34.2%)	44 (36.7%)	0.783
**Procedural characteristics**				
Oxygenation pre-ECMO				
FiO_2_ (%)	100 (80–100)	90 (80–100)	100 (80–100)	0.233
Horowitz index (mmHg)	77.1 (63.1–107.1)	77.6 (60.1–105.2)	76.8 (63.2–109.0)	0.828
D(A-a)O_2_ (mmHg)	531 (419–592)	492 (416–579)	542 (418–595)	0.369
Duration of MV before ECMO (days)	1.3 (0.3–5.0)	2.2 (0.2–7.6)	1.1 (0.3–3.5)	0.133
Prone positioning before ECMO	26 (16.5%)	7 (18.4%)	19 (15.8%)	0.708
Acute renal failure	50 (31.6%)	13 (34.2%)	37 (30.8%)	0.696
Scores				
SOFA score	14.0 (11.0–16.0)	13.0 (11.0–15.0)	14.0 (11.0–16.8)	0.146
APACHE II score	26.0 (21.8–32.0)	24.0 (22.8–28.3)	27.0 (20.3–32.0)	0.364
RESP score	1 (− 2.0–2.0)	0 (− 3.0–2.0)	1.0 (− 2.0–2.0)	0.702
Causes of ARDS				
Pneumonia	116 (73.4%)	33 (86.8%)	83 (69.2%)	0.032
Aspiration	15 (9.5%)	2 (5.3%)	13 (10.8%)	0.307
Inhalation injury	1 (0.6%)	0 (0.0%)	1 (0.8%)	0.572
Drowning	2 (1.3%)	0 (0.0%)	2 (1.7%)	0.423
Autoimmune injury	10 (6.3%)	2 (5.3%)	8 (6.7%)	0.757
Sepsis	10 (6.3%)	1 (2.6%)	9 (7.5%)	0.283
Pancreatitis	1 (0.6%)	0 (0.0%)	1 (0.8%)	0.572
Other injuries	3 (1.9%)	0 (0.0%)	3 (2.5%)	0.325
Pulmonary pathogen spectrum				
Bacterial	62 (39.2%)	16 (42.1%)	46 (38.3%)	0.678
Viral	30 (19.0%)	13 (34.2%)	17 (14.2%)	0.006
Fungal	27 (17.1%)	14 (36.8%)	13 (10.8%)	< 0.001
*Pneumocystis jirovecii*	11 (7.0%)	7 (18.4%)	4 (3.3%)	0.001

*APACHE II* Acute Physiology and Chronic Health Evaluation, *ARDS* acute respiratory distress syndrome, *BMI* body mass index, *COPD* chronic obstructive pulmonary disease, *CAD* coronary artery disease, *D(A-a)O*_*2*_ alveolar-arterial gradient of oxygen concentration, *ECMO* extracorporeal membrane oxygenation, *FiO*_*2*_ fraction of inspired oxygen, *LTOT* long-term oxygen therapy, *MV* mechanical ventilation, *RESP* Respiratory Extracorporeal Membrane Oxygenation Survival Prediction, *SOFA* Sequential Organ Failure Assessment. Categorical variables are presented as frequency (percentages). Continuous variables are presented as median (IQR)

Thirty-eight patients (24.1%) received PP during ECMO therapy. No relevant complications (e.g. decannulation) occurred during the positioning procedures. Patients with PP during ECMO support had a higher rate of pre-existing chronic renal failure and pneumonia-induced ARDS. Patients in the prone group displayed a different pulmonary pathogen spectrum (more viral and fungal infections, especially *Pneumocystis jirovecii*, Table 1). Survival prediction scores (SOFA, APACHE II, and RESP) did not differ between both groups. PP before ECMO initiation was performed in 16.5% of the patients in both groups.

On average, the first PP during ECMO support was performed after 1.7 (0.5–5.0) days on ECMO support, with 2.0 (1.0–3.0) PP manoeuvres performed per patient. Average PP duration was 19.5 (16.8–20.8) h (Additional file 1, table E1).

### Procedural characteristics and outcome

Patients with PP during ECMO support showed higher PEEP levels from day 4 and higher plateau pressures from day 4 to 8 (Additional file 1, figure E1). There was no difference in driving pressures as well as in tidal volumes. However, patients with PP during ECMO support showed less spontaneous breathing on day 5 and day 8 to 10.

**Fig. 1:**
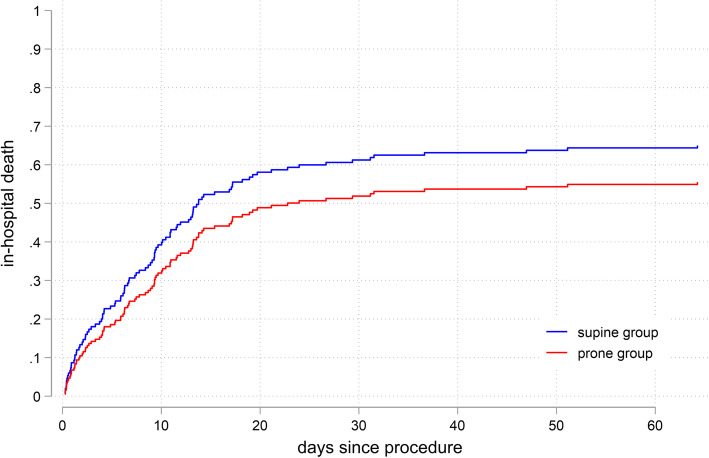
In-hospital death of ECMO patients with vs. without prone positioning during ECMO. The Fine-Gray model for in-hospital death (SHR 0.77, *p* = 0.21, cumulative incidence of 60-day death 55% vs. 64%). ECMO, extracorporeal membrane oxygenation

There were no differences in ECMO weaning rate (47.4% vs. 46.7%, *p* = 0.940), and ICU or hospital survival (36.8% vs. 36.7%, respectively, *p* = 0.984) between the prone and the supine groups (Table 2). Cumulative incidences of 60-day in-hospital death were 55% and 64% for the prone and supine groups, respectively (*p* = 0.207, Fig. 1).

**Table 2:** Outcome and procedural characteristics

	All (*n* = 158)	Prone (*n* = 38)	Supine (*n* = 120)	*p* value
Weaning successful	74 (46.8%)	18 (47.4%)	56 (46.7%)	0.940
30-day survival	65 (41.1%)	18 (47.4%)	47 (39.2%)	0.371
ICU survival	58 (36.7%)	14 (36.8%)	44 (36.7%)	0.984
Hospital survival	58 (36.7%)	14 (36.8%)	44 (36.7%)	0.984
ECMO duration (days)	6.6 (3.9–11.1)	10.7 (6.7–17.1)	5.9 (2.5–9.2)	< 0.001
ICU length of stay (days)	13.3 (9.1–23.1)	18.0 (12.0–31.1)	12.3 (7.2–20.1)	0.002
MV duration (days)	12.0 (6.8–21.1)	18.7 (11.8–30.9)	9.9 (4.4–18.7)	< 0.001
Haemodialysis	62 (39.2%)	16 (42.1%)	46 (38.3%)	0.678
Tracheostomy	61 (38.6%)	20 (52.6%)	41 (34.2%)	0.042

*ECMO* extracorporeal membrane oxygenation, *ICU* intensive care unit, *MV* mechanical ventilation. Categorical variables are presented as frequency (percentages). Continuous variables are presented as median (IQR)

### Propensity score matching analysis

**Fig. 2:**
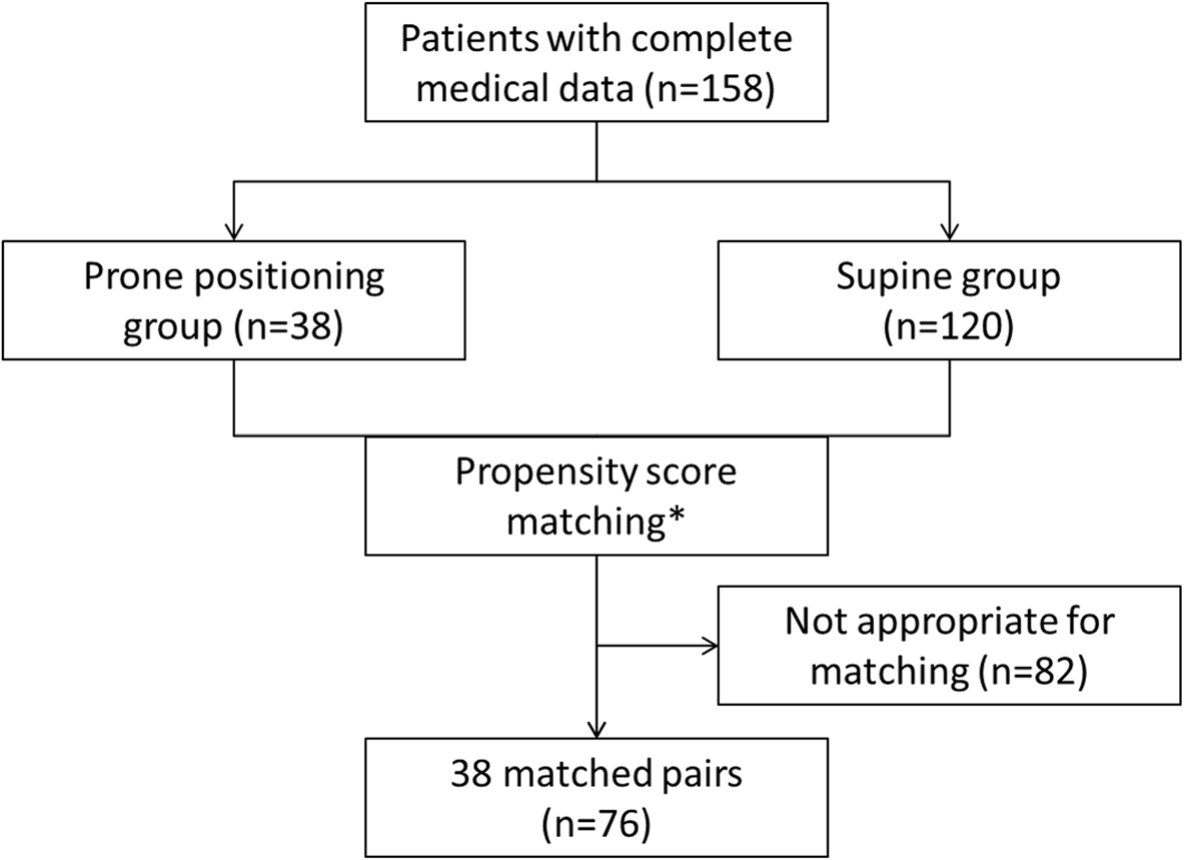
Propensity score matched pair patient assignment. *Matching was performed for age, sex, SOFA score, the duration of MV before ECMO, and performance of prior PP before ECMO. ECMO, extracorporeal membrane oxygenation; MV, mechanical ventilation; PP, prone positioning; SOFA, Sequential Organ Failure Assessment

Thirty-eight propensity score matched pairs (76 patients) with similar baseline characteristics could be analysed (Fig. 2, see also Additional file 1, table E2). Successful ECMO weaning rate was 47.4% vs. 44.7% (*p* = 0.818) in patients with and without PP during ECMO support, respectively. Furthermore, there was no difference in survival between both groups (36.8% vs. 36.8%, *p* = 1.0). Cumulative incidences of 60-day in-hospital death were 58% and 65% for the prone and supine groups, respectively (*p* = 0.482, Additional file 1, figure E1).

### Prognostic factors

Underlying lung fibrosis, status of immunosuppression, and aspiration were associated with death, whereas proof of bacterial infections was associated with survival (Table 3). Moreover, a high proportion of spontaneous breathing in the first 10 days was strongly associated with survival. In multivariate analysis, only underlying lung fibrosis (odds ratio 0.15 [95% CI 0.0–0.7]) and a high proportion of spontaneous breathing in the first 10 days (odds ratio 20.0 [95% CI 5.4–73.5]) were independent predictors for death and survival, respectively.

**Table 3:** Prognostic factors

	Dead (*n* = 100)	Survivors (*n* = 58)	*p* value
Lung fibrosis	16 (16.0%)	2 (3.4%)	0.017
Immunosuppression	43 (43.0%)	14 (24.1%)	0.017
Causes of ARDS: aspiration	13 (13.0%)	2 (3.4%)	0.048
Proof of pulmonary bacterial infection	31 (31.0%)	31 (53.4%)	0.005
Proportion of spontaneous breathing (d1–10, %)	50.0 (0–80)	80.0 (60–80)	< 0.001

Shown are the parameters with association to survival or death in patients with severe acute respiratory distress syndrome and extracorporeal membrane oxygenation support in a univariate analysis

Categorical variables are presented as frequency (percentages). Continuous variables are presented as median (IQR)

**Fig. 3:**
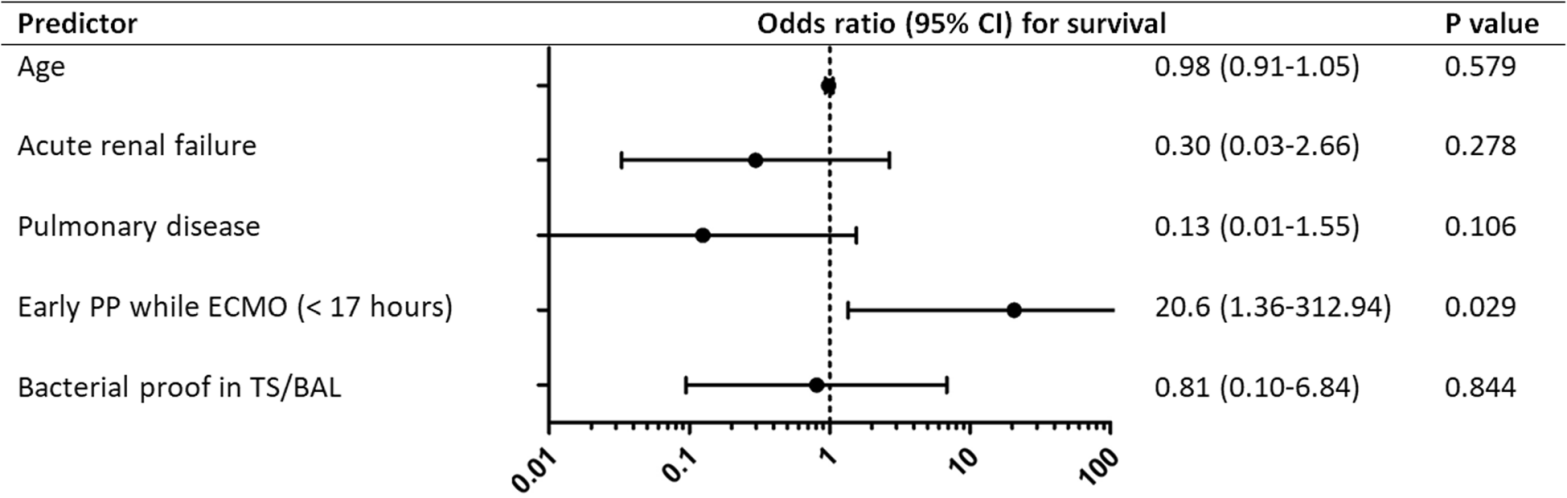
Multivariate analysis of survival in patients treated with prone positioning during ECMO. Early initiation of prone positioning (< 17 h) was an independent predictor for survival in patients with prone positioning during ECMO. BAL, bronchoalveolar lavage; ECMO, extracorporeal membrane oxygenation; PP, prone positioning; TS, tracheal secretions

In patients with PP, higher age, acute renal failure, and underlying pulmonary disease were associated with death. Proof of pulmonary bacterial infection and timing of the first PP after ECMO initiation were associated with survival in a univariate analysis (Additional file 1, table E4). In a multivariate analysis, only early initiation of PP (< 17 h) was associated with survival (odds ratio 20.6 [95% CI 1.4–312.9], Fig. 3).

Optimal cutoff value for duration from ECMO initiation to first PP was calculated using ROC analysis (AUC = 0.789) and Youden’s Index. Highest sensitivity and specificity for beneficial survival were achieved for initiation of PP in < 17 h. Next to this optimal cutoff, a clinical cutoff of 1 day (24 h) also was associated with improved survival (*p* = 0.005).

**Fig. 4:**
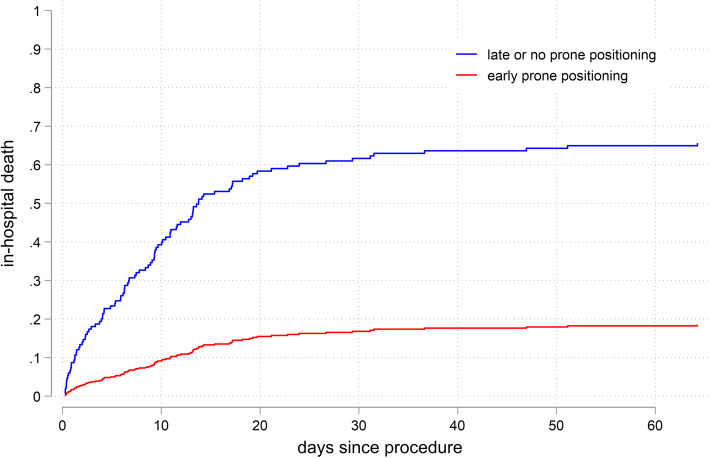
In-hospital death of ECMO patients depending on performance of early prone positioning during ECMO. The Fine-Gray model for in-hospital death, early (cutoff 17 h) vs. late or no prone positioning during ECMO (SHR 0.19, *p* = 0.03, cumulative incidence of 60-day death 18% vs. 65%). ECMO, extracorporeal membrane oxygenation

Patients treated with early PP during ECMO (*n* = 11) showed a superior survival to patients treated with late PP or without PP during ECMO support (81.8% vs. 33.3%). Cumulative incidences of 60-day in-hospital death were 18% for the early PP group and 65% for the late and no PP group, respectively (*p* = 0.027, Fig. 4). Also, in a separate comparison of patients with late PP as well as patients without PP, early PP showed superior survival rates (81.8% vs. 18.5% and 36.7%, *p* < 0.001 and *p* = 0.003, respectively).

Patients in the early PP group were younger than patients with late or without PP during ECMO support (40.0 vs. 56.0 years, *p* = 0.004). The groups did not differ concerning vasoactive support or in SOFA and APACHE II scores at the time of ECMO implantation. Moreover, there was no difference in the SOFA score between both groups in the first 3 days (Additional file 1, table E8). The RESP score of the patients with early PP was higher (2.0 (1.0–6.0) vs. 0 (− 2.0–2.0), *p* = 0.025, Additional file 1, table E5). The RESP score without including age was 3 (2.0–6.0) vs. 2 (0–4.0), *p* = 0.096).

## Discussion

Prone positioning has shown to improve survival in non-ECMO ARDS patients [5]. There is sparse data on PP in ARDS patients with VV ECMO support. We therefore retrospectively analysed a large cohort of ECMO patients suffering from severe ARDS treated with or without PP at our centre. Our results do not indicate an overall survival benefit for PP during ECMO support per se. However, timing of PP may be crucial when designing future studies.

In comparison to previous PP studies, technical execution of PP in this analysis showed favourable characteristics. Beginning of PP after ECMO initiation was earlier than in other studies (1.7 vs. 6 or 9 days, respectively) [6, 10]. Moreover, the average duration of each performed PP was longer (19.5 h) and more PP manoeuvres were performed per patient (2.0) than described before [10, 11, 22]. This is especially important, as the survival benefit for PP in ARDS without ECMO support shown by Guerin et al. was achieved with long PP periods (17 h) [5]. Patients treated with PP in our patient collective showed increased PEEP and plateau pressure levels but still remained in the recommended limits of the ELSO guideline [3]. As intended by the treating medical team, driving pressure was kept below 15 cmH_2_O, as high driving pressures are strongly associated with increased mortality [23]. Furthermore, no differences in driving pressure were found between both groups.

Patients with PP during ECMO showed a reduced rate of spontaneous breathing compared to patients without PP, despite the fact that neuromuscular blocking agents were not used on a routine basis during PP periods. However, it seems reasonable that PP patients might have been on deeper sedation levels than patients in the supine group. In contrast to this, the ELSO guidelines recommend an early reduction of sedation levels and a switch to spontaneous breathing after 24 to 48 h after ECMO initiation [3]. Furthermore, low proportions of spontaneous breathing episodes were associated with a higher mortality. However, this only allows hypothesis generating, since causality between a reduced rate of spontaneous breathing and increased mortality cannot be proven in this analysis and could also be an expression of higher disease severity. Nevertheless, the reduced rate of spontaneous breathing in patients with PP should be considered in the discussion of benefits and disadvantages of this additional treatment.

Our results are in contrast to the study of Guervilly et al. Their retrospective study of additional PP showed an encouraging survival benefit [24]. Survival rate in the PP group was markedly higher than in the supine group (30-day survival 71% vs. 43%). In terms of age, sex, and PP manoeuvres performed per patient, the cohort of Guervilly et al. and our patients did not differ. However, our patients were sicker than those of Guervilly and co-workers (predicted mortality by SOFA score approx. 55% vs. 35% [16]) and showed a much lower rate of prior PP before ECMO (17% vs. 64%). Furthermore, Guervilly et al. reported deep sedation and routine use of neuromuscular blocking agents during PP which is in contrast to our approach. To compare our findings with those from Guervilly et al., we used the same matching parameters for propensity score matching, which did not alter our findings.

Timing of PP was an independent predictor of survival in our cohort. Early initiation of PP after ECMO cannulation was strongly associated with improved survival. A beginning of PP in less than 1 day (cutoff < 17 h via Youden’s Index) in comparison to late or no PP showed a strong survival benefit (82% vs. 33%). This finding is in line with the study protocol of the PROSEVA trial [5], where the survival benefit for PP in non-ECMO ARDS patients was achieved with an early beginning of PP (initiated in average 36 h after beginning of mechanical ventilation). This association suggests that an early beginning of PP after initiation of ECMO support could be an important factor for survival, which requires further investigation.

Because of the retrospective design of this study, the reasons why patients were treated with PP or not, or received early or late PP, cannot be pinned down. Patients receiving early PP were younger, but they did not differ in terms of haemodynamic stability and showed no difference regarding the SOFA and APACHE II scores. Patients of the early PP group showed a higher RESP score (2.0 vs. 0), indicating a certain difference in predicted mortality rate (35% vs. 50%). Nevertheless, the factor age could have influenced the team’s decision-making for or against early PP. Interestingly, in the early PP group, in contrast to the whole PP group, a higher rate of spontaneous breathing within the first 10 days was observed (not significant), which could be one factor that may improve survival rate for early PP.

From a theoretical standpoint, there are many positive effects of additional PP in patients receiving ECMO support, like improving oxygenation and lung compliance as well as reducing ventilator-induced lung injury [6–8]. In clinical practice, patient-safety concerns often prevent prone positioning during ECMO therapy, even though feasibility and safety have been demonstrated in several studies.

In this retrospective analysis, PP at any time was not associated with improved survival per se. However, our results indicate that a very early initiation of PP therapy (within 1 day after cannulation) could be beneficial. No complications related to PP were detected. In consideration of the retrospective design of this study, we think that a randomised controlled trial is imperatively needed for further evaluation of PP in ECMO patients. Considering the pros and cons of a PP therapy, PP should not be withheld from ARDS patients requiring ECMO support. Our data suggest that PP should be initiated very early in the clinical course.

### Limitations

This is a retrospective observational study and therefore contains the risk of selection and reporting bias. Another limitation is the small sample size of only 38 patients with PP and 76 patients in the matched pair analysis, respectively. Moreover, this is a single-centre report and specific processes may influence the presented results. The same internal standard operating procedures applied to the entire treating physician team. However, the indication for performing PP during ECMO support was on basis of the treating ECMO physician and therefore was not standardised. Despite using propensity score matching for outcome analysis, this among other factors might be remaining confounders that we did not control for. Together, due to these limitations, our findings should be considered as hypothesis generating and should not prompt clinical decision-making.

## Conclusion

This retrospective analysis did not reveal an overall survival benefit associated with PP in patients with ARDS requiring ECMO support. However, a subgroup analysis suggested that early initiation of PP may improve survival and should be considered in the design of a randomised controlled trial for further evaluation.

## Supplementary information

**Additional file 1.** Prone positioning in severe ARDS requiring extracorporeal membrane oxygenation – Online Data Supplement. Description: supplemental figure E1-E2 and table E1-E8.

## Data Availability

The datasets used and/or analysed during the current study are available from the corresponding author on reasonable request.

## Abbreviations

APACHE II: Acute Physiology and Chronic Health Evaluation
ARDS: Acute respiratory distress syndrome
BAL: Bronchoalveolar lavage
BMI: Body mass index
CAD: Coronary artery disease
COPD: Chronic obstructive pulmonary disease
D(A-a)O_2_: Alveolar-arterial gradient of oxygen concentration
ECMO: Extracorporeal membrane oxygenation
FiO_2_: Fraction of inspired oxygen
ICU: Intensive care unit
LTOT: Long-term oxygen therapy
MV: Mechanical ventilation
PEEP: Positive end expiratory pressure
PP: Prone positioning
RESP: Respiratory Extracorporeal Membrane Oxygenation Survival Prediction
SOFA: Sequential Organ Failure Assessment
TS: Tracheal secretions
VV: Veno-venous
