# Supplementary material for: Prone positioning in severe ARDS requiring extracorporeal membrane oxygenation
Source: Crit Care. 2020 Jul 8;24:397. doi: 10.1186/s13054-020-03110-2 (PMC7341706; doi:10.1186/s13054-020-03110-2)
Supplement: Supplementary file 1 — Additional file 1. Prone positioning in severe ARDS requiring extracorporeal membrane oxygenation – Online Data Supplement. Description: supplemental figure E1-E2 and table E1-E8. [file 13054_2020_3110_MOESM1_ESM.docx]

## Online Data Supplement

**Prone positioning in severe ARDS requiring extracorporeal membrane oxygenation**

Jonathan Rilinger, Viviane Zotzmann, Xavier Bemtgen, Carin Schumacher, Paul M Biever, Daniel Duerschmied, Klaus Kaier, Peter Stachon, Constantin von zur Mühlen, Manfred Zehender, Christoph Bode, Dawid L Staudacher, Tobias Wengenmayer

Categorical variables are presented as frequency (percentages). Continuous variables are presented as median (IQR).


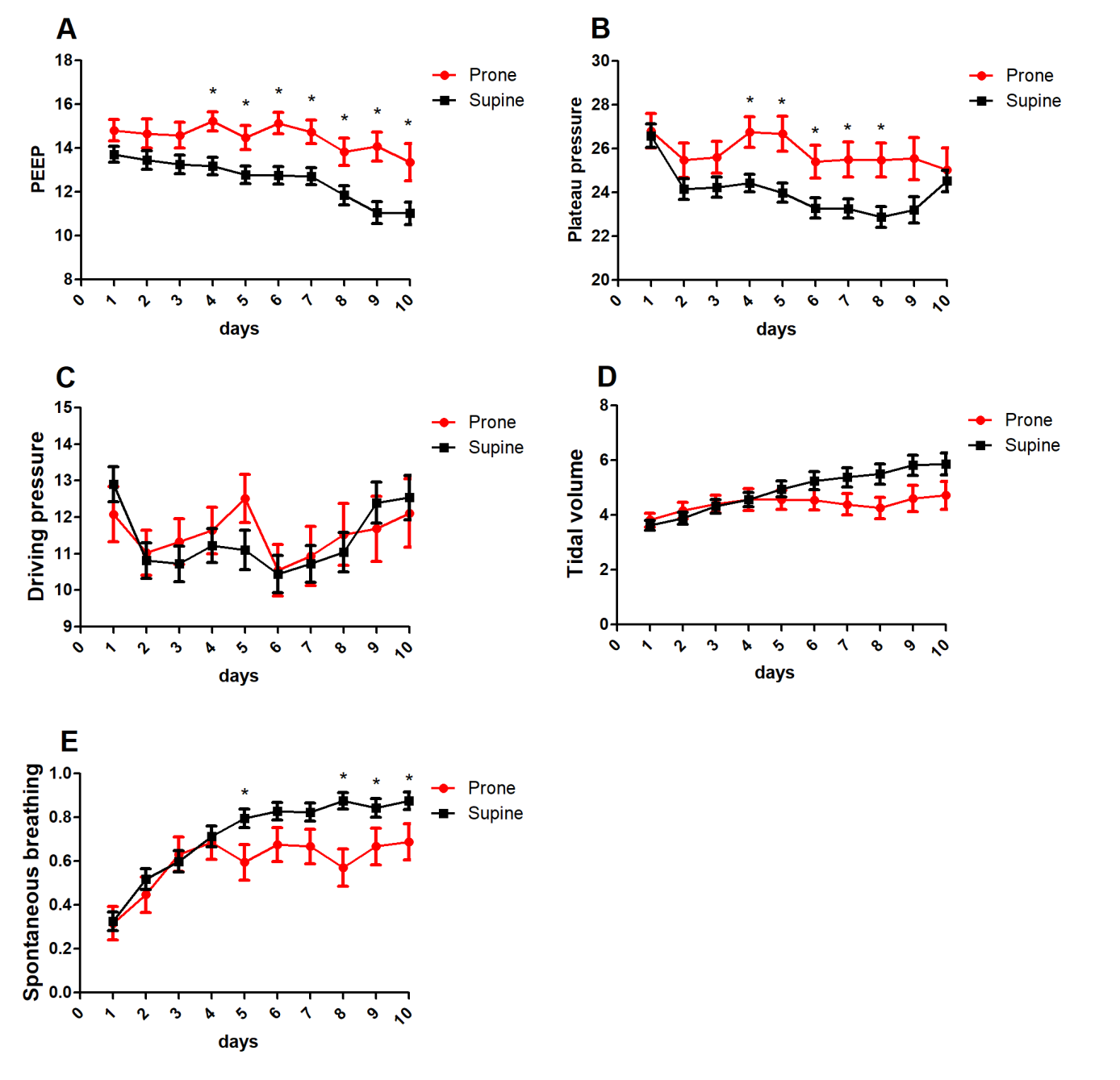


### Figure E1. Mechanical ventilation under ECMO support with vs. without prone positioning

A) PEEP was increased in patients with prone positioning after day 3. B) Plateau pressure was increased in patients with prone positioning on day 4 to 8. C and D) There was no difference in driving pressure and tidal volume between patients with and without prone positioning. E) Patients with prone positioning showed less spontaneous breathing on day 5 and day 8 to 10.

(Pressure levels of mechanical ventilation are shown in cmH_2_O, tidal volume is shown in ml/kg (idealised) body weight.)

* = p<0.05. *ECMO: extracorporeal membrane oxygenation; PEEP: positive end expiratory pressure;* *PP: prone positioning.*

### Figure E2. In-hospital death of ECMO patients with vs. without prone positioning during ECMO in the propensity score matched cohort

Fine-Gray model for in-hospital death (SHR 0.81, p=0.482, cumulative incidence of 60 day death 58% vs. 65%).

*ECMO: extracorporeal membrane oxygenation.*

### Table E1. Details of prone positioning during ECMO support

| **Count of PP** | **Rate of executed PP** | **Duration of PP (h)** |
| --- | --- | --- |
| 1 | 38 (100%) | 19.7 (17.9-20.9) |
| 2 | 27 (71.1%) | 20.0 (19.0-21.0) |
| 3 | 17 (44.7%) | 18.0 (13.3-21.3) |
| 4 | 8 (21.1%) | 16.6 (10.7-18.4) |
| 5 | 5 (13.2%) | 16.8 (10.4-19.9) |
| 6 | 2 (5.3%) | 17.1 (14.4 and 19.8)* |
| 7 | 2 (5.3%) | 13.3 (12.8 and 13.8)* |

*ECMO: extracorporeal membrane oxygenation; h: hours; PP: Prone positioning.*

*no IQR available in case of two values.

Median (IQR) duration of all 99 performed PP procedures: 19.5 (16.8-20.8) hours.

### Table E2. Baseline characteristics and outcome of propensity score matched cohort

|  | **All**  **(n=76)** | **Prone**  **(n=38)** | **Supine**  **(n=38)** |
| --- | --- | --- | --- |
| Age (y) | 54.0 (40.3-63.5) | 51.5 (38.5-64.0) | 55.5 (44.0-62.5) |
| Sex (male) | 60 (78.9%) | 28 (73.7%) | 32 (84.2%) |
| Underlying pulmonary disease | 30 (39.5%) | 12 (31.6%) | 18 (47.4%) |
| Comorbidities |  |  |  |
| Chronic renal failure | 9 (11.8%) | 6 (15.8%) | 3 (7.9%) |
| Chronic   haemodialysis | 2 (2.6%) | 1 (2.6%) | 1 (2.6%) |
| Immunosuppression | 26 (34.2%) | 13 (34.2%) | 13 (34.2%) |
| Oxygenation pre ECMO |  |  |  |
| FiO_2_ (%) | 100 (71.3-100) | 90 (80-100) | 100 (63.8-100) |
| Horowitz index   (mmHg) | 78.8  (64.3-107.6) | 77.6  (60.2-105.2) | 82.2  (64.4-118.8) |
| D(A-a)O_2_ (mmHg) | 496 (373-589) | 493 (417-579) | 526 (327-597) |
| Duration of MV before  ECMO (d) | 2.2 (0.2-6.7) | 2.2 (0.2-7.6) | 1.7 (0.1-6.5) |
| Prone positioning before ECMO | 16 (21.1%) | 7 (18.4%) | 9 (23.7%) |
| Acute renal failure | 22 (28.9%) | 13 (34.2%) | 9 (23.7%) |
| Scores |  |  |  |
| SOFA score | 13.0 (11.0-15.0) | 13.0 (11.0-15.0) | 12.0 (8.8-16.0) |
| APACHE II score | 24.0 (18.3-28.8) | 24.0 (22.8-28.3) | 22.0 (14.0-29.3) |
| RESP score | 0 (-2.0-2.0) | 0 (-3.0-2.0) | 0 (-2.0-2.0) |
| Causes of ARDS |  |  |  |
| Pneumonia | 65 (85.5%) | 33 (86.8%) | 32 (84.2%) |
| Aspiration | 4 (5.3%) | 2 (5.3%) | 2 (5.3%) |
| Other injuries | 7 (9.2%) | 3 (7.9%) | 4 (10.5%) |
|  |  |  |  |
| Weaning successful | 35 (46.1%) | 18 (47.4%) | 17 (44.7%) |
| 30d-survival | 34 (44.7%) | 18 (47.4%) | 16 (42.1%) |
| ICU survival | 28 (36.8%) | 14 (36.8%) | 14 (36.8%) |
| Hospital survival | 28 (36.8%) | 14 (36.8%) | 14 (36.8%) |
| ECMO duration (d) | 8.5 (4.7-16.5) | 10.7 (6.7-17.1) | 5.4 (3.2-11.2) |
| MV duration (d) | 16.8 (9.4-29.3) | 18.7 (11.9-30.9) | 11.3 (6.6-20.6) |
| ICU length of stay (d) | 15.8 (10.5-29.6) | 18.0 (12.0-31.1) | 13.1 (8.2-24.0) |
| Proportion of spontaneous breathing (d1-10, %) | 75.0  (34.4-90.0) | 60.0  (35.6-90.0) | 82.9  (31.3-92.5) |

*APACHE II score: Acute Physiology And Chronic Health Evaluation; ARDS: acute respiratory distress syndrome; D(A-a)O_2_: alveolar-arterial gradient of oxygen concentration; ECMO: extracorporeal membrane oxygenation; FiO2: fraction of inspired oxygen; ICU: intensive care unit; MV: mechanical ventilation; RESP score: Respiratory Extracorporeal Membrane Oxygenation Survival Prediction; SOFA score: Sequential Organ Failure Assessment.*

### Table E3. Univariate prognostic analysis of patients with severe ARDS and ECMO support

|  | **Dead**  **(n=100)** | **Survivors**  **(n=58)** | **P value** |
| --- | --- | --- | --- |
| Age (y) | 56.0 (44.3-65.0) | 51.0 (40.8-61.2) | 0.304 |
| Sex (male) | 64 (64.0%) | 42 (72.4%) | 0.278 |
| BMI (kg/m²) | 24.9 (23.6-27.8) | 24.3 (22.0-29.7) | 0.518 |
| Underlying pulmonary disease | 38 (38.0%) | 17 (29.3%) | 0.269 |
| COPD | 7 (7.0%) | 4 (6.9%) | 0.980 |
| Asthma | 8 (8.0%) | 2 (3.4%) | 0.257 |
| Lung fibrosis | 16 (16.0%) | 2 (3.4%) | 0.017 |
| Cystic fibrosis | 6 (6.0%) | 1 (1.7%) | 0.208 |
| LTOT | 9 (9.0%) | 2 (3.4%) | 0.186 |
| Pulmonary  hypertension | 5 (5.0%) | 1 (1.7%) | 0.299 |
| Comorbidities |  |  |  |
| Nicotine abuse | 27 (27.0%) | 20 (34.5%) | 0.321 |
| Hypertension | 26 (26.0%) | 23 (39.7%) | 0.074 |
| Diabetes mellitus | 15 (15.0%) | 9 (15.5%) | 0.930 |
| CAD | 12 (12.0%) | 8 (13.8%) | 0.744 |
| Chronic renal failure | 6 (6.0%) | 6 (10.3%) | 0.320 |
| Chronic haemodialysis | 1 (1.0%) | 1 (1.7%) | 0.695 |
| Liver cirrhosis/hepatitis | 14 (14.0%) | 3 (5.2%) | 0.084 |
| Immunosuppression | 43 (43.0%) | 14 (24.1%) | 0.017 |
| Oxygenation pre ECMO |  |  |  |
| FiO_2_ (%) | 100 (80-100) | 100 (75-100) | 0.558 |
| Horowitz index (mmHg) | 76.2 (62.3-108.3) | 77.6 (63.7-107.0) | 0.826 |
| D(A-a)O_2_ (mmHg) | 535 (420-596) | 528 (380-580) | 0.407 |
| MV duration before ECMO (d) | 1.4 (0.2-5.2) | 1.3 (0.3-4.3) | 0.827 |
| Prone positioning before ECMO | 18 (18.0%) | 8 (13.8%) | 0.492 |
| Acute renal failure | 27 (27.0%) | 23 (31.6%) | 0.099 |
| Causes of ARDS |  |  |  |
| Pneumonia | 72 (72.0%) | 44 (75.9%) | 0.596 |
| Aspiration | 13 (13.0%) | 2 (3.4%) | 0.048 |
| Inhalation injury | 0 (0%) | 1 (1.7%) | 0.188 |
| Drowning | 2 (2.0%) | 0 (0%) | 0.278 |
| Autoimmune injury | 5 (5.0%) | 5 (8.6%) | 0.368 |
| Sepsis | 6 (6.0%) | 4 (6.9%) | 0.823 |
| Pancreatitis | 0 (0%) | 1 (1.7%) | 0.188 |
| Other injuries | 2 (2.0%) | 1 (1.7%) | 0.903 |
| Pulmonary pathogen spectrum |  |  |  |
| Bacterial | 31 (31.0%) | 31 (53.4%) | 0.005 |
| Viral | 17 (17.0%) | 13 (22.4%) | 0.403 |
| Fungal | 19 (19.0%) | 8 (13.8%) | 0.402 |
| Pneumocystis   jirovecii | 7 (7.0%) | 4 (6.9%) | 0.980 |
| Proportion of spontaneous breathing  (d1-10, %) | 50.0 (0-80) | 80.0 (60-80) | <0.001 |

*ARDS: acute respiratory distress syndrome; BMI: body mass index; COPD: chronic obstructive pulmonary disease; CAD: coronary artery disease; D(A-a)O2: alveolar-arterial gradient of oxygen concentration; ECMO: extracorporeal membrane oxygenation; FiO2: fraction of inspired oxygen; LTOT: long-term oxygen therapy; MV: mechanical ventilation.*

### Table E4. Univariate prognostic analysis of patients treated with prone positioning during ECMO

|  | **Dead**  **(n=24)** | **Survivors**  **(n=14)** | **P value** |
| --- | --- | --- | --- |
| Age (y) | 57.0 (49.3-57.0) | 45.5 (32.8-52.3) | 0.068 |
| Sex (male) | 17 (70.8%) | 11 (78.6%) | 0.601 |
| Underlying pulmonary disease | 10 (41.7%) | 2 (14.3%) | 0.080 |
| COPD | 1 (4.2%) | 0 (0%) | 0.439 |
| Asthma | 1 (4.2%) | 0 (0%) | 0.439 |
| Lung fibrosis | 5 (20.8%) | 0 (0%) | 0.067 |
| Cystic fibrosis | 2 (8.3%) | 0 (0%) | 0.267 |
| LTOT | 1 (4.2%) | 0 (0%) | 0.439 |
| Pulmonary  hypertension | 0 (0%) | 0 (0%) | - |
| Comorbidities |  |  |  |
| Nicotine abuse | 6 (25.0%) | 3 (21.4%) | 0.803 |
| Hypertension | 8 (33.3%) | 2 (14.3%) | 0.198 |
| CAD | 3 (12.5%) | 2 (14.3%) | 0.875 |
| Chronic renal failure | 4 (16.7%) | 3 (14.3%) | 0.846 |
| Chronic haemodialysis | 1 (4.2%) | 0 (0%) | 0.439 |
| Liver cirrhosis/hepatitis | 2 (8.3%) | 1 (7.1%) | 0.896 |
| Immunosuppression | 9 (37.5%) | 4 (28.6%) | 0.576 |
| Oxygenation pre ECMO |  |  |  |
| FiO_2_ (%) | 87.5 (80-100) | 95.0 (71.3-100) | 0.917 |
| Horowitz index (mmHg) | 75.9 (58.2-103.7) | 81.5 (68.3-105.9) | 0.750 |
| D(A-a)O_2_ (mmHg) | 482 (420-572) | 511 (364-585) | 0.802 |
| MV duration before ECMO (d) | 2.2 (0.2-7.0) | 2.5 (0.2-8.7) | 0.893 |
| Duration ECMO to first PP | 2.7 (0.9-6.8) | 0.5 (0.3-2.3) | 0.003 |
| ECMO to 1.PP < 1 d | 6 (25.0%) | 10 (71.4%) | 0.005 |
| ECMO to 1.PP < 0.71 d | 2 (8.3%) | 9 (64.3%) | <0.001 |
| Count of PP during ECMO | 2.0 (1.0-3.0) | 2.0 (1.0-3.3) | 0.988 |
| Prone positioning before ECMO | 6 (25.0%) | 1 (7.1%) | 0.171 |
| Acute renal failure | 6 (25.0%) | 7 (50.0%) | 0.117 |
| Causes of ARDS |  |  |  |
| Pneumonia | 21 (87.5%) | 12 (85.7%) | 0.875 |
| Aspiration | 1 (4.2%) | 1 (7.1%) | 0.692 |
| Inhalation injury | 0 (0%) | 0 (0%) | - |
| Drowning | 0 (0%) | 0 (0%) | - |
| Autoimmune injury | 2 (8.3%) | 0 (0%) | 0.267 |
| Sepsis | 2 (8.3%) | 1 (7.1%) | 0.185 |
| Pancreatitis | 0 (0%) | 0 (0%) | - |
| Other injuries | 0 (0%) | 0 (0%) | - |
| Pulmonary pathogen spectrum |  |  |  |
| Bacterial | 7 (29.2%) | 9 (64.3%) | 0.034 |
| Viral | 8 (33.3%) | 5 (35.7%) | 0.881 |
| Fungal | 10 (41.7%) | 4 (28.6%) | 0.420 |
| Pneumocystis   jirovecii | 5 (20.8%) | 2 (14.3%) | 0.615 |
| Proportion of spontaneous breathing  (d1-10, %) | 53.4 (30.0-85.6) | 75.0 (57.5-90.0) | 0.113 |

*ARDS: acute respiratory distress syndrome; COPD: chronic obstructive pulmonary disease; CAD: coronary artery disease; D(A-a)O2: alveolar-arterial gradient of oxygen concentration; ECMO: extracorporeal membrane oxygenation; FiO2: fraction of inspired oxygen; LTOT: long-term oxygen therapy; MV: mechanical ventilation.*

### Table E5. Baseline characteristics and outcome of early PP vs. no or late PP

|  | **All**  **(n=158)** | **Early PP**  **(n=11)** | **Late or no PP**  **(n=147)** | **P value** |
| --- | --- | --- | --- | --- |
| Age (y) | 54.5  (41.8-64.0) | 40.0  (26.0-51.0) | 56.0  (45.0-65.0) | 0.004 |
| Sex (male) | 106 (67.1%) | 9 (81.8%) | 97 (66.0%) | 0.281 |
| BMI (kg/m²) | 24.7  (22.9-28.8) | 24.1  (20.5-24.7) | 24.7  (23.4-29.4) | 0.119 |
| Underlying pulmonary disease | 55 (34.8%) | 3 (27.3%) | 52 (35.4%) | 0.586 |
| COPD | 11 (7.0%) | 0 (0%) | 11 (7.5%) | 0.347 |
| Asthma | 10 (6.3%) | 0 (0%) | 10 (6.8%) | 0.371 |
| Lung fibrosis | 18 (11.4%) | 0 (0%) | 18 (12.2%) | 0.218 |
| Cystic fibrosis | 7 (4.4%) | 1 (9.1%) | 6 (4.1%) | 0.436 |
| LTOT | 11 (7.0%) | 0 (0%) | 11 (7.5%) | 0.347 |
| Pulmonary  hypertension | 6 (3.8%) | 0 (0%) | 6 (4.1%) | 0.495 |
| Comorbidities |  |  |  |  |
| Nicotine abuse | 47 (29.7%) | 3 (27.3%) | 44 (29.9%) | 0.852 |
| Chronic renal failure | 12 (7.6%) | 1 (9.1%) | 11 (7.5%) | 0.846 |
| Chronic   haemodialysis | 2 (1.3%) | 0 (0%) | 2 (1.4%) | 0.697 |
| Liver cirrhosis/  hepatitis | 17 (10.8%) | 2 (18.2%) | 15 (10.2%) | 0.410 |
| Immunosuppression | 57 (36.1%) | 2 (18.2%) | 55 (37.4%) | 0.200 |
| Oxygenation pre ECMO |  |  |  |  |
| FiO_2_ (%) | 100 (80-100) | 90 (75-100) | 100 (80-100) | 0.456 |
| Horowitz index   (mmHg) | 77.1  (63.1-107.1) | 77.8  (65.9-105.6) | 76.9  (62.1-107.3) | 0.803 |
| D(A-a)O_2_ (mmHg) | 531 (419-592) | 528 (373-559) | 532 (419-593) | 0.285 |
| Duration of MV before  ECMO (d) | 1.3 (0.3-5.0) | 2.1 (0.2-8.6) | 1.3 (0.3-4.3) | 0.403 |
| Prone positioning before ECMO | 26 (16.5%) | 1 (9.1%) | 25 (17.0%) | 0.495 |
| Acute renal failure | 50 (31.6%) | 5 (45.5%) | 45 (30.6%) | 0.307 |
| Haemodynamic |  |  |  |  |
| Noradrenaline (µg/min/kg) | 2.8 (0.8-5.5) | 2.7 (0.8-6.2) | 2.8 (0.8-5.5) | 0.790 |
| Suprarenine (µg/min/kg) | 0.0 (0.0-0.0) | 0.0 (0.0-0.0) | 0.0 (0.0-0.0) | 0.251 |
| Lactat (mmol/L) | 1.8 (1.1-4.5) | 1.3 (1.0-2.3) | 1.9 (1.1-4.7) | 0.431 |
| Scores |  |  |  |  |
| SOFA score | 14.0  (11.0-16.0) | 14.0  (12.0-15.0) | 14.0  (11.0-16.0) | 0.813 |
| APACHE-II score | 26.0  (21.8-32.0) | 25.0  (23.0-35.0) | 26.0  (21.0-32.0) | 0.662 |
| RESP score | 1 (-2.0-2.0) | 2.0 (1.0-6.0) | 0 (-2.0-2.0) | 0.025 |
| RESP score   (without age) | 2 (0-4.0) | 3 (2.0-6.0) | 2 (0-4.0) | 0.096 |
| Causes of ARDS |  |  |  |  |
| Pneumonia | 116 (73.4%) | 9 (81.8%) | 107 (72.8%) | 0.513 |
| Aspiration | 15 (9.5%) | 1 (9.1%) | 14 (9.5%) | 0.962 |
| Other | 27 (17.1%) | 1 (9.1%) | 26 (17.7%) | 0.735 |
| Pulmonary pathogen spectrum |  |  |  |  |
| Bacterial | 62 (39.2%) | 9 (81.8%) | 53 (36.1%) | 0.003 |
| Viral | 30 (19.0%) | 3 (27.3%) | 27 (18.4%) | 0.468 |
| Fungal | 27 (17.1%) | 2 (18.2%) | 25 (17.0%) | 0.920 |
| Pneumocystis   jirovecii | 11 (7.0%) | 1 (9.1%) | 10 (6.8%) | 0.774 |
|  |  |  |  |  |
| Weaning successful | 74 (46.8%) | 9 (81.8%) | 65 (44.2%) | 0.016 |
| 30d-survival | 65 (41.1%) | 9 (81.8%) | 56 (38.1%) | 0.004 |
| ICU survival | 58 (36.7%) | 9 (81.8%) | 49 (33.3%) | 0.001 |
| Hospital survival | 58 (36.7%) | 9 (81.8%) | 49 (33.3%) | 0.001 |
| ECMO duration (d) | 6.6 (3.9-11.1) | 6.7 (5.1-11.7) | 6.6 (3.7-11.0) | 0.878 |
| MV duration (d) | 12.0  (6.8-21.1) | 17.1  (10.3-31.0) | 12.0  (6.1-21.1) | 0.147 |
| ICU length of stay (d) | 13.3  (9.1-23.1) | 17.6  (11.4-28.7) | 13.2  (8.7-23.0) | 0.162 |
| Proportion of spontaneous breathing (d1-10, %) | 70 (30-90) | 80 (60-90) | 70 (30-90) | 0.271 |

*APACHE II score: Acute Physiology And Chronic Health Evaluation; ARDS: acute respiratory distress syndrome; BMI: body mass index; COPD: chronic obstructive pulmonary disease; D(A-a)O2: alveolar-arterial gradient of oxygen concentration; ECMO: extracorporeal membrane oxygenation; FiO2: fraction of inspired oxygen; ICU: intensive care unit; LTOT: long-term oxygen therapy; MV: mechanical ventilation; RESP score: Respiratory Extracorporeal Membrane Oxygenation Survival Prediction; SOFA score: Sequential Organ Failure Assessment.*

### Table E6. Severe complications on VV ECMO support

| **Complication type** | **Quantity (in 158 patients)** |
| --- | --- |
| Severe bleeding (pulmonary, gastrointestinal, cerebral) | 23 (3 fatal) |
| Severe thrombosis (pump head) | 3 (1 fatal) |
| Perforation (pericardial tamponade, mediastinal) | 2 (both non-fatal) |
| Vascular lesion | 1 |
| Accidental decannulation | 2 |
| Air suction in ECMO system | 1 (fatal) |

*ECMO: extracorporeal membrane oxygenation; VV: veno-venous.*

**Table E7. Mode of death**

| **Cause of death** | **Quantity (in 100 patients)** |
| --- | --- |
| Circulatory failure | 32 |
| ECMO/Ventilator weaning failure* | 38 |
| Hypoxia | 7 |
| Neurological impairment* | 15 |
| Lethal ECMO related complications† | 2 |
| Other Organ failure | 6 |

* withdrawal of care; † failure of the pump head due to thrombosis, air suction in ECMO system for dilated tracheotomy. *ECMO: extracorporeal membrane oxygenation.*

### Table E7. Cut off determination for early prone positioning and survival

| Time PP to first ECMO (d) | Sensitivity | Specificity | Youden-Index |
| --- | --- | --- | --- |
| 0.116 | 0.07 | 1.00 | 0.07 |
| 0.163 | 0.14 | 1.00 | 0.14 |
| 0.222 | 0.14 | 0.96 | 0.10 |
| 0.259 | 0.21 | 0.96 | 0.17 |
| 0.286 | 0.29 | 0.96 | 0.24 |
| 0.382 | 0.43 | 0.96 | 0.39 |
| 0.465 | 0.50 | 0.96 | 0.46 |
| 0.490 | 0.57 | 0.96 | 0.53 |
| 0.600 | 0.57 | 0.92 | 0.49 |
| **0.715** | **0.64** | **0.92** | **0.56** |
| 0.760 | 0.64 | 0.88 | 0.52 |
| 0.802 | 0.64 | 0.83 | 0.48 |
| 0.844 | 0.64 | 0.79 | 0.43 |
| 0.888 | 0.71 | 0.79 | 0.51 |
| 0.986 | 0.71 | 0.75 | 0.46 |
| 1.078 | 0.71 | 0.71 | 0.42 |
| 1.344 | 0.71 | 0.67 | 0.38 |
| 1.672 | 0.71 | 0.63 | 0.34 |
| 1.908 | 0.71 | 0.58 | 0.30 |
| 2.122 | 0.71 | 0.54 | 0.26 |
| 2.233 | 0.79 | 0.54 | 0.33 |
| 2.534 | 0.79 | 0.50 | 0.29 |
| 2.884 | 0.86 | 0.50 | 0.36 |
| 3.208 | 0.86 | 0.46 | 0.32 |
| 3.792 | 0.86 | 0.42 | 0.27 |
| 4.448 | 0.86 | 0.38 | 0.23 |
| 4.833 | 0.86 | 0.33 | 0.19 |
| 5.076 | 0.86 | 0.29 | 0.15 |
| 5.347 | 0.93 | 0.29 | 0.22 |
| 5.549 | 0.93 | 0.25 | 0.18 |
| 6.396 | 1.00 | 0.25 | 0.25 |
| 7.187 | 1.00 | 0.21 | 0.21 |
| 9.150 | 1.00 | 0.17 | 0.17 |
| 11.450 | 1.00 | 0.13 | 0.13 |
| 12.334 | 1.00 | 0.08 | 0.08 |
| 13.267 | 1.00 | 0.04 | 0.04 |
| 14.667 | 1.00 | 0.00 | 0.00 |

Youden-Index = Sensitivity + Specificity - 1.

Highest sensitivity and specificity was achieved for 0.71 days = 17.04 hours.

*ECMO: extracorporeal membrane oxygenation; PP: prone positioning.*

### Table E8. Prospective SOFA score of early PP vs. late or no PP

|  | **All**  **(n=158)** | **Early PP**  **(n=11)** | **Late or no PP**  **(n=147)** | **P value** |
| --- | --- | --- | --- | --- |
| d0 | 14 (11-16) | 14 (12-15) | 14.0 (11-16) | 0.813 |
| d1 | 12 (9.5-14) | 11 (9-14) | 12 (10-14) | 0.481 |
| d3 | 12 (9-14) | 13 (9-15) | 12 (9-14) | 0.846 |
| d7 | 11 (8.8-14) | 7.5 (6-15) | 11 (9-14) | 0.260 |
| d10 | 10 (8-13) | 8 (4.5-10) | 11 (8-13) | 0.068 |

*SOFA score: Sequential Organ Failure Assessment; PP: prone positioning*
